# Instantaneous Sinapisms

**Published:** 1876-11

**Authors:** 


INSTANTANEOUS SINAPISMS.—Dr. Rigabert describes a sinapism invented by M. Vincent, a chemist at Saintes, which, he says, for promptitude and certainty surpass Rigollot's papers. These last are apt to deteriorate by keeping, and at all events require the aid of water to produce their effects. M. Vincent's procedure is as follows: Into a tube, open at the end, five centimetres in length, and having a calibre of half a centimetre, he pours a certain quantity of freshly prepared essence of mustard; the tube is corked and hermetically closed, and is wrapped up in a piece of paper of tolerable consistence of the size of one of Rigollot's sinapisms. When it has to be used, some drops of the essence are poured on the paper, which is applied as an ordinary sinapism. The effect is instantaneous and certain; and by pouring on the paper the contents of two tubes at the same time, vesication can be produced.—*Bull. de Therap.*

